# Long-gap esophageal atresia: is native esophagus preservation always possible?

**DOI:** 10.3389/fped.2024.1450378

**Published:** 2024-08-29

**Authors:** G. M. Treccarichi, V. Di Benedetto, G. Loria, M. G. Scuderi

**Affiliations:** ^1^ Pediatric Surgery Unit, Department of Medical and Surgical Sciences and Advanced Technologies, G. F. Ingrassia, University of Catania, AOU Policlinico “G. Rodolico-San Marco”, Catania, Italy; ^2^ Unit of Pathology, Department of Human Pathology in Adulthood and Childhood Gaetano Barresi, University of Messina, Messina, Italy

**Keywords:** esophageal atresia, long gap esophageal atresia, esophageal replacement, esophageal repair, esophageal salvage, anastomotic stricture, gastroesophageal reflux

## Abstract

**Introduction:**

Esophageal atresia (EA) is a congenital defect that causes esophageal discontinuity, often with an associated tracheo-esophageal fistula (TEF) in 70%–90% of cases. When the distance between esophageal ends precludes primary anastomosis, it results in long gap esophageal atresia (LGEA), complicating the surgical management. This study retrospectively reviewed LGEA cases from the past decade, treated with the goal of preserving the native esophagus, comparing surgical techniques and outcomes with current literature.

**Materials and methods:**

The data of patients treated for LGEA between 2013 and 2024 were collected from medical charts, focusing on patients treated with the preservation of their native esophagus.

**Results:**

Ten patients were enrolled for this study. All of them had a gap between the esophageal ends equal to or greater than three vertebral bodies. Four patients (40%) underwent a delayed primary anastomosis (DPA) procedure, while the remaining six (60%) underwent a traction staged repair. All patients were treated with open surgery. The follow-up period extended from 3 months to 10 years.

**Conclusion:**

Preserving the native esophagus in patients with LGEA is a challenging but feasible goal, with delayed primary anastomosis and traction techniques playing key roles. We advocate for the preservation of the native esophagus as the preferred approach for ensuring a high quality of life for patients, as it helps to avoid severe long-term complications associated with esophageal substitution.

## 1 Introduction

Esophageal atresia (EA) is a congenital esophageal discontinuity resulting from incomplete development of the middle fraction of the esophagus, leading to proximal esophageal obstruction. The incidence of EA is approximately 1:4,000 live births, with a male predominance, making it one of the most common congenital anomalies of the esophagus. In 70%–90% of patients, EA is associated with a tracheo-esophageal fistula (TEF). This anatomic anomaly is thought to arise from incomplete division of the esophago-tracheal diverticulum of the foregut during the fourth week of gestation.

Based on the presence and/or proximity of the TEF, EA is classified into five anatomical subtypes (1).

Around 50% of EA cases occur alongside other systems anomalies, such as those observed in VACTERL syndrome (Vertebral, Anorectal, Cardiac, Tracheal, Esophageal, Renal and Limb anomalies) (2). According to the degree of distance between the upper and lower ends of the atretic esophagus, the terms short gap (SGEA) and long gap esophageal atresia (LGEA) are applied.

The definition of LGEA is still not clear due to the lack of consensus among the scientific community.

LGEA, accounting for less than 10% of all EA cases, presents management challenges due to limited evidence and consensus on surgical approaches (3).

In a significant proportion of LGEA cases, primary anastomosis is difficult to achieve, requiring alternative surgical techniques to restore esophageal continuity. Cervical esophagostomy is among the potential maneuvers considered during the initial management phase of the patient, but it should be avoided if preservation of the native esophagus is desired, since it is likely to compromise the possibility of conducting a primary esophago-esophagostomy in the future and requires esophageal replacement (4). Esophageal replacement can be pursued through gastric pull-up (GPU), gastric tube reconstruction (GTR), jejunal or colon interpositions. Due the high incidence of complications and long-term side effects associated with these replacement procedures, a lot of research has focused on developing surgical strategies to preserve the infant's native esophagus, such as extrathoracic esophageal stretching, proximal and distal myotomies, bougienage, and the newer esophageal magnetic anastomosis (5).

Delayed primary anastomosis (DPA) is strongly preferred as the initial approach in the management of LGEA and is supported by the American Pediatric Surgery Association recommendations and by the 2019 consensus conference of the European Reference Network for Rare Inherited Congenital Anomalies (ERNICA) (3, 6). The delayed approach allows the growth of the esophagus. This growth is likely induced by oral secretions in the proximal pouch and distally by gastroesophageal reflux (7).

Esophageal growth can be relatively rapidly induced by traction suture. Indeed, one of the most frequently employed approaches for traction staged repair is the Foker technique, which offers a two-stage open surgical procedure to preserve the native esophagus, involving traction on the atretic esophageal segments to promote growth in the first stage, followed by esophageal anastomosis in the second stage (8).

The aim of this study was to retrospectively review LGEA cases treated in our center over the past 11 years, focusing on surgical techniques employed to restore native esophageal anatomy and comparing outcomes with the current literature.

## 2 Materials and methods

### 2.1 Data collection

We retrospectively reviewed the medical charts of patients diagnosed with LGEA from March 2013 to early 2024 at our tertiary pediatric surgery unit. Overall, 38 patients were diagnosed with EA, of whom 12 were classified as having LGEA. In our cohort, LGEA was defined as an esophageal gap greater than 3 vertebral bodies on chest x-ray or cases where a tension-free primary anastomosis could not be achieved at the time of the first surgical attempt.

The inclusion criteria were the preservation of the native esophagus, leading to the enrollment of 10 patients in this study. Two out of 12 infants with LGEA were excluded: one underwent gastric interposition, while the other died without attempted repair due to associated malformations.

In the case where esophageal preservation could not be pursued, the patient was affected by CHARGE syndrome with choanal atresia and a severe interventricular defect. The EA presented an extremely wide esophageal gap, greater than 6 vertebral bodies, with a very small distal esophageal stump. Given the patient's comorbidities and esophageal anatomy, a one-stage esophageal surgical repair with a gastric pull-up was considered the best treatment in this particular case.

Data on patients’ demographic and clinical characteristics were collected, including gender, gestational age, birth weight, associated anomalies (Table 1). Intraoperative gap length, age at primary repair, length of hospital stay (LOS), and short- and medium-term postoperative complications such as respiratory distress, recurrent esophageal strictures, and the need for anti-reflux surgery were also recorded. The type of EA was classified according to the VOGT criteria (Table 2) (9).

**Table 1:** Patients’ demographics and clinical characteristics.

	Study cohort
Number of patients	*n* = 10
Male sex	60% (*n* = 6)
Mean gestational age (weeks)	34 (range: 26–38 weeks)
Mean delivery weight (gr)	1,985 gr (range: 1,150–2,670 gr)
Associated cardiovascular anomalies	20% (*n* = 2)
Associated anorectal malformation/cloaca	20% (*n* = 2)

Our cohort consisted in ten patients with LGEA, with a slight male predominance (60%). Gestational age at birth ranged from 26 to 38 weeks, with a median value of 34 weeks. The mean birth weight was 1,985 grams, ranging from 1,150 to 2,670 grams. Twenty percent of the babies were diagnosed with cardiovascular anomalies, and another 20% had an associated anorectal malformation.

**Table 2:** Classification of patients according to vogt criteria for EA.

	Study cohort
Type I	40% (*n* = 4)
Type II	0% (*n* = 0)
Type III	60% (*n* = 6)

The majority of our patients were affected by Type III EA, presenting with a distal tracheoesophageal fistula (60%). Four out of ten patients (40%) were diagnosed with isolated EA without any tracheoesophageal fistula (Type I EA).

Follow-up included clinical evaluation and additional paraclinical investigations.

### 2.2 Preoperative management

**Figure 1:**
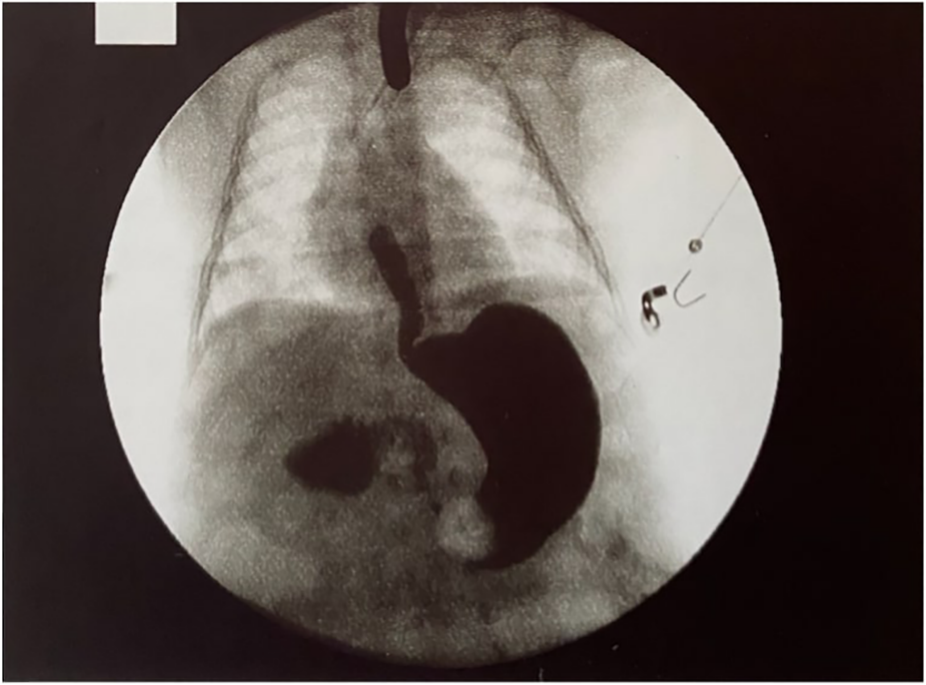
X-Ray evaluation of the distance between upper and lower pouch.

In almost all patients with LGEA, a gastrostomy was performed to establish stable enteral feeding access, while in some cases, nutritional care was provided through parenteral feeding via a central venous catheter. Once the gastrostomy had healed, gap studies were conducted to evaluate the distance between the upper and lower esophageal pouches (Figure 1). The use of a Replogle tube connected to continuous suction helped prevent recurrent oropharyngeal aspiration by draining the upper pouch. Furthermore, maintaining the patient in an anti-Trendelenburg position, with the head elevated above the trunk, helped prevent aspiration.

### 2.3 Surgical technique

**Figure 2:**
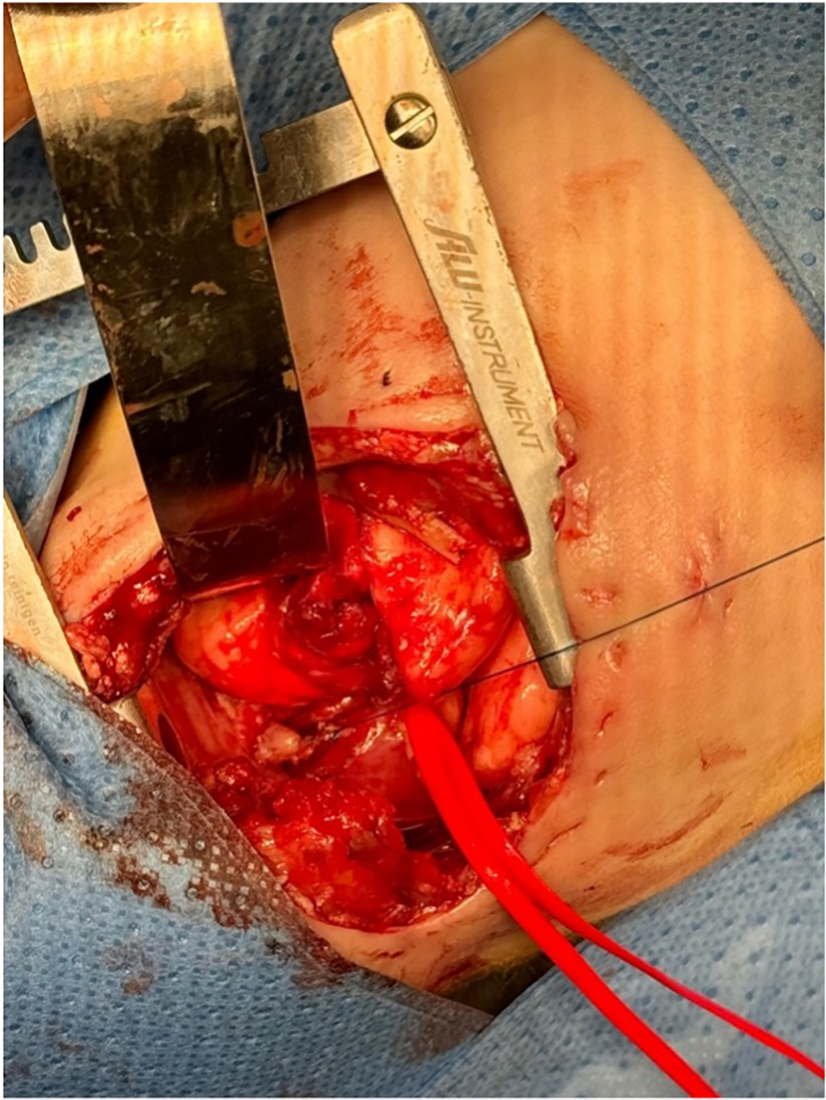
Proximal esophageal segment previously tractioned.

The first surgical step involved closing the fistula in cases with TEF. Access to the thorax was gained through a right thoracotomy at the fourth intercostal space by dividing the external and internal intercostal muscles. Subsequently, the fistula was divided and closed using 5/0 Vicryl sutures. The possibility of achieving a primary anastomosis was not ruled out until the anatomy was inspected during the thoracotomy. If the distance between the esophageal pouches did not allow for a primary anastomosis, mobilization of the upper and lower esophageal segments with traction was required to promote esophageal growth (Figure 2). Traction-induced mobilization was achieved using Gore-Tex patches, which were anchored to the internal intercostal muscles, one intercostal space below the ends of the esophageal pouches. Ultimately, a gastrostomy was performed for enteral feeding purposes.

**Figure 3:**
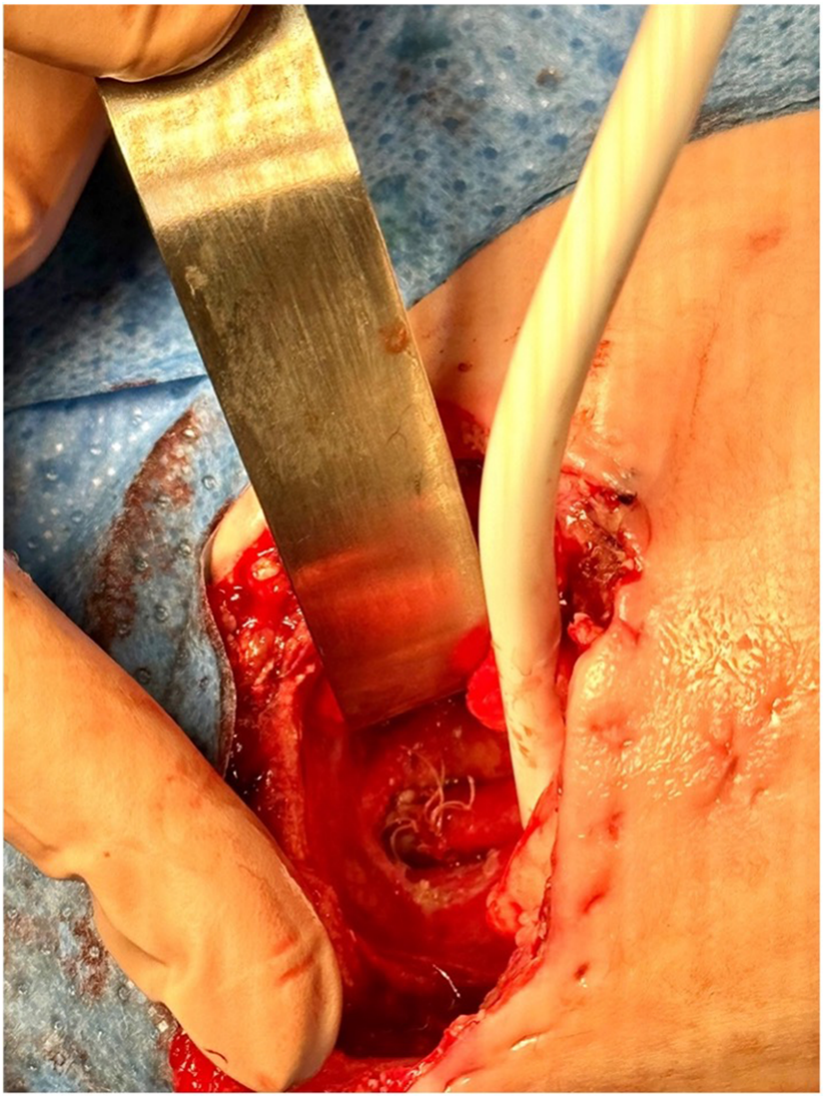
Esophago-esophageal anastomosis.

Repair was typically performed when a significant reduction in the gap length was achieved, as identified by x-ray imaging showing the proximal pouch below the thoracic inlet. By reopening the prior thoracotomy and mobilizing the esophageal pouches, the anastomosis was performed using multiple circumferential single stitches over an 8/6 French nasogastric tube (Figure 3). The gastrostomy was then closed. The nasogastric tube was left in place for approximately 7–10 days after surgery until an x-ray contrast study was performed to assess the anastomosis. A chest drain tube was placed and was typically removed within a week.

Regarding the surgical technique, we consider the use of Gore-Tex patches crucial for achieving high levels of tension on the esophageal stumps while avoiding damage to the intercostal musculature to which they are anchored. Additionally, oblique cutting of the distal esophageal stump, rather than horizontal cutting, facilitated the creation of a wider anastomosis and helped prevent severe postoperative strictures. It is worth noting that, to facilitate the approximation of the stumps, dissection should be balanced according to their vascularization. Intense skeletonization to free the upper stump from adhesions is better tolerated due to the blood supply provided by the thyroid vessels, whereas excessive skeletonization of the distal stump could lead to ischemic damage and subsequent postoperative stenosis.

### 2.4 Postoperative care and complications

Postoperative ventilation under deep sedation was administered in all cases. x-ray imaging was performed approximately 24 h after surgery to monitor for pneumothorax. Complications were analyzed in terms of respiratory distress, esophageal stricture requiring dilatation, and gastroesophageal reflux disease requiring corrective surgery.

## 3 Results

### 3.1 Patients’ cohort and surgery technical notes

Among our 12 patients treated for LGEA, two were excluded: the first one died before the procedure of esophago-esophageal anastomosis due to associated malformations; the other patient underwent gastric interposition. In this latter case, esophageal gap had an extreme width, greater than 6 vertebral bodies with a minute distal esophageal stump. The patient was diagnosed with CHARGE syndrome, with choanal atresia and a severe interventricular defect associated to LGEA. He required several surgeries for comorbidities, so a one-stage esophageal surgical repair was considered the best treatment in this selected case.

Ten patients were finally included in this study. According to the Vogt classification, 40% were diagnosed with type I EA, and 60% with type III (a, b), including 6 males (60%) and 4 females (40%), resulting in a male-to-female ratio of 1.5:1. Seven patients (70%) underwent a gastrostomy procedure, while the remaining patients (30%) were treated without gastrostomy. All patients underwent esophageal gap length measurement at the time of the first thoracotomy, and in all cases, the length of the gap was three or more vertebral bodies.

Four patients (40%) underwent only a delayed primary anastomosis (DPA) procedure, while the remaining six (60%) underwent a traction staged repair. All patients were treated with open surgery. The median age at the time of the esophago-esophageal anastomosis was 86 days (range: 9–192 days), and the average length of hospital stay (LOS) was 165 days (range: 55–413 days).

In our cohort, the maximum gap length was observed in an extremely premature infant with Fallot tetralogy, whose x-ray revealed a distance slightly greater than 4 vertebral bodies. After establishing a traction system, a second look was conducted after about 12 weeks, but the gap was still too wide to allow a tension-free anastomosis. Delayed anastomosis was successfully achieved only after 192 days of traction.

In one patient, instead, stumps traction before DPA consisted of only 9 days duration. This case involved a newborn with multiple associated malformations and an intraoperative gap length of about 4 centimeters at the time of TEF closure. The upper pouch was enmeshed with multiple adhesions, whose careful liberation reduced the gap to about 1 centimeter. Aiming for a tension-free anastomosis, a traction system was established. On the 9th day, a surgical procedure was needed for a complication after choanal atresia correction. Considering the immediate pre-operative chest x-ray and the necessity of sedation for the ENT pathology, our team took the opportunity to check the feasibility of esophageal repair, which was then accomplished.

### 3.2 Post-operative management

During the postoperative period, 3 (30%) patients suffered from respiratory distress. Gastroesophageal reflux disease (GERD) was observed in 3 patients (30%) and was treated with a Nissen fundoplication. One patient died 208 days after surgical correction due to associated anomalies not related to the repair. The follow-up period, ranging from 3 months to 10 years, was conducted through x-ray esophagograms. During this period, anastomotic strictures were observed in 7 patients (70%) and were treated with endoscopic esophageal dilation. Among them, approximately 70% became symptomatic at the beginning of the weaning phase. All patients with anastomotic strictures underwent at least one endoscopy to calibrate the esophagus and check the mucosa. Multiple dilations were required in 3 patients.

**Figure 4:**
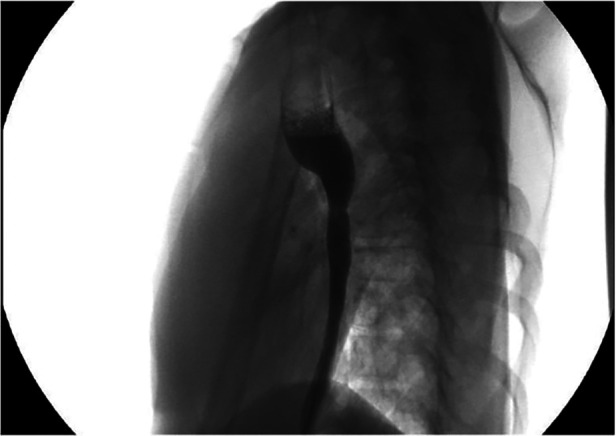
Esophagogram of a patient 10 years post-surgery.

**Figure 5:**
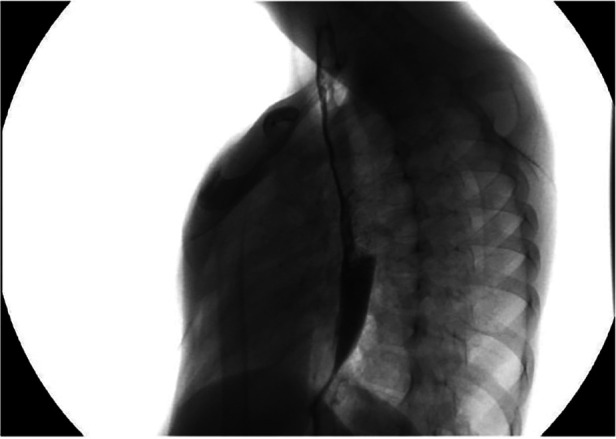
Esophagogram of a patient 5 years post-surgery.

Our results demonstrated that, despite the complications observed in the short, medium, and long-term, all patients resumed oral feeding and did not require additional surgical interventions (Figures 4, 5).

## 4 Discussion

The aim of this study is to support the evidence that, although the management of LGEA represents a significant surgical challenge, preserving the native esophagus is not only a possible treatment but should be the primary goal in LGEA management whenever feasible. This objective aligns with the consensus recommendations from the American Pediatric Surgery Association and the ERNICA, both advocating for delayed primary anastomosis (DPA) when the gap between the esophageal ends is too extensive for immediate anastomosis. Our findings corroborate these guidelines, demonstrating that DPA and internal traction techniques can effectively promote esophageal growth and enable subsequent anastomosis, achieving a high success rate.

Comparing our results with other surgical management approaches for LGEA in the literature, our experience contrasts with reports such as the study by Jensen et al., where 21% of patients with a significant gap length were treated with a reverse gastric tube or gastric transposition, without considering any lengthening procedure (10). Similarly, Huh et al*.* compared the outcomes between two groups of patients, one of which was treated via esophago-gastric tube anastomosis when the gap length was equal to or greater than four vertebral bodies, attesting that in cases with wide gap, esophageal reconstruction is performed (11). In Gallo et al. study, the definition of LGEA was similar to ours, but patients were treated with gastric pull-up (GPU) or jejunal interposition; moreover, one patient underwent GPU on the first day of life due to the impossibility of performing an immediate primary anastomosis (12).

Among our cases, one patient presented a particularly complicated situation due to a proximal esophageal pouch that was initially too short. According to the reported literature, in this complicated situation, a cervical esophagostomy should have been performed, subjecting the patient to an almost certain need for esophageal replacement at a later time. However, after applying traction to the pouch, it extended to the level of the left pulmonary apex, making the creation of the anastomosis possible, although under tension.

The reason our study focuses on preserving the native esophagus is due to the numerous, yet avoidable, complications associated with esophageal replacement. Specifically, the use of a gastric conduit may result in gastric stasis, delayed gastric emptying, or difficulties in swallowing, which could require eating small and frequent meals. Additionally, the stomach occupying a significant portion of the chest cavity may compress intrathoracic organs. The most feared complications are esophagitis and metaplasia. Jejunal conduits pose risks of graft ulcers or complete graft necrosis due to reduced blood supply, potentially leading to graft perforation and mediastinitis. Finally, colon conduits may lead to diverticulum formation, conduit redundancy causing delayed emptying, and the development of adenoma (12, 13).

On the other hand, the alternative approach of awaiting spontaneous esophageal growth presents inevitable disadvantages, including the necessity for gastrostomy placement, prolonged hospital stays, and the risk of aspiration pneumonia requiring continuous upper pouch suction. Likewise, the internal traction technique presents critical issues, such as the need for patients to remain intubated and sedated in the NICU. The median length of hospital stay (LOS) was 165 days, reflecting the complexity and prolonged nature of LGEA management.

The surgical approach, primarily open surgery, remains the standard in our institution, although minimally invasive techniques are gaining popularity. The Italian Society of Videosurgery in Infancy (SIVI) guidelines suggest that the thoracoscopic approach offers several technical and other advantages over open. thoracotomy, such as decreased postoperative intubation time, shorter time to start and regain oral feeding, and decreased duration of postoperative analgesia (14).

Almost all patients experienced postoperative complications, with a high incidence of anastomotic strictures (70%) and gastroesophageal reflux disease (GERD) (30%). However, this outcome is not uncommon and has been reported as a common long-term complication following repair surgery in patients with LGEA (15, 16). Upadhyaya et al. reported a stricture rate of 75% in patients with a wide gap length, which was similar to our findings (17). The high rate of anastomotic strictures necessitated frequent endoscopic dilations, a well-known and accepted part of the postoperative management for these patients. GERD, observed in nearly half of our patients, required surgical intervention in the form of Nissen fundoplication, highlighting the need for vigilant postoperative monitoring and management to address these common issues.

## 5 Conclusion

The surgical management of esophageal atresia, with or without TEF, represents a technical and clinical challenge for pediatric surgeons. The preservation of the native esophagus in patients with LGEA is a challenging yet achievable goal. According to our experience, esophageal preservation is feasible with a gap length of up to four vertebral bodies. Delayed primary anastomosis and traction techniques play crucial roles in this process. While postoperative complications are common, they are manageable with appropriate surgical and medical interventions. Despite the limited number of patients in our cohort, we achieved a high success rate in preserving the native esophagus, even though we have previously resorted to esophageal replacement techniques. Our findings support the continued use of these techniques and underscore the importance of a multidisciplinary approach in managing LGEA to optimize patient outcomes, while the use of techniques that do not preserve the native esophagus should be considered only as a rescue therapy (18).

This study suggests that, even in severe cases of LGEA, an attempt to preserve the native esophagus should always be made to avoid all the complications associated with esophageal replacement.

Further studies with larger cohorts are needed to refine these techniques, improve long-term results, and reduce the incidence of postoperative complications for patients with LGEA.
